# Influence of delay between dental bleaching with 35% hydrogen peroxide and orthodontic brackets on the bond strength at the enamel/adhesive interface

**DOI:** 10.4317/jced.55719

**Published:** 2019-05-01

**Authors:** Antônio-Augusto-Lima de Almeida, Darlon-Martins Lima, Adriana-de Fátima-Vasconcelos Pereira, Soraia-de Fátima-Carvalho Sousa, Cláudia-Maria-Coêlho Alves

**Affiliations:** 1 Master of Science in Dentistry - Post-Graduate Program in Dentistry, Federal University of Maranhão, São Luís, MA, Brasil; 2 PhD, Post-Graduate Program in Dentistry. Federal University of Maranhão, São Luís, MA, Brasil

## Abstract

**Background:**

The aim of this study was to evaluate the influence of waiting time between the bleaching with 35% hydrogen peroxide and orthodontic brackets bonding on shear bond strength (SBS) in enamel.

**Material and Methods:**

Eighty bovine teeth were randomly divided into four groups (G): G1(negative control) and G2, G3 and G4 (experimental groups). The experimental groups were submitted to bleaching. Prior to orthodontic brackets bonding to enamel the procedure was adopted different waiting times, as follows: G2 (1 day); G3 (7 days) and G4 (14 days). It was performed enamel etching (30s), washing water (30s), application of adhesive system followed by photoactivation (20s). A thin layer of composite resin was placed between the adhesive and the brackets. The applied pressure was measured by tensiometer (300N/40s). The composite resin was light-cured (40s). After 24 hours the shear test was held (0.5mm/min). To compare the SBS it was used ANOVA one-way followed by Tukey test (α = 0.05). The Adhesive Remaining Index (ARI) was analyzed using the Kruskal-Wallis test.

**Results:**

The SBS values were significantly lower in G2 (15.51 MPa) and G3 (17.77 MPa) compared to G1 (30.14 MPa) and G4 (28.50 MPa) (*p*<0.05). The ARI revealed significant difference between the G3 and the other groups (*p*<0.05).

**Conclusions:**

It was concluded that the bond strength in enamel in the interfaces/adhesive system/composite resin/orthodontic brackets was more effective 14 days after the bleaching with 35% hydrogen peroxide.

** Key words:**Dental materials, teeth bleaching, orthodontic brackets.

## Introduction

Tooth bleaching has become a safe option, mainly in patients who have stained teeth due to the consumption of fluorosis or consumption of certain beverages (1). Whitening treatment can be performed at home or carried out at office (2). Home bleaching is usually done with 10 to 22%, carbamide peroxide whereas office bleaching is done with 35% hydrogen peroxide. In-office whitening is often chosen due to its advantages of convenience and speed (3). Orthodontic treatment has become a popular option among patients of all ages. Its effectiveness depends on the bonding of orthodontic brackets to enamel, among other factors. Tooth whitening can interfere with the effectiveness of adhesive procedures (3,4). Some studies suggest that patients wait at least 7 or 14 days after bleaching before undergoing bracket bonding, to ensure the restoration of normal bond strength values (3,5). Although the negative influence of bleaching on bond strength has been confirmed (6–8), there is no consensus on the ideal interval between tooth whitening and bracket bonding to obtain effective adhesion of the brackets to enamel (5,9-13). Therefore, the aim of this study was to evaluate the influence of the time interval between bleaching and bracket bonding on the shear bond strength (SBS) of orthodontic brackets bonded to enamel. The null hypothesis was that the delay after bleaching would have no influence on the SBS at the adhesive/enamel interface.

## Material and Methods

-Selection and preparation of specimens

**Figure 1:**
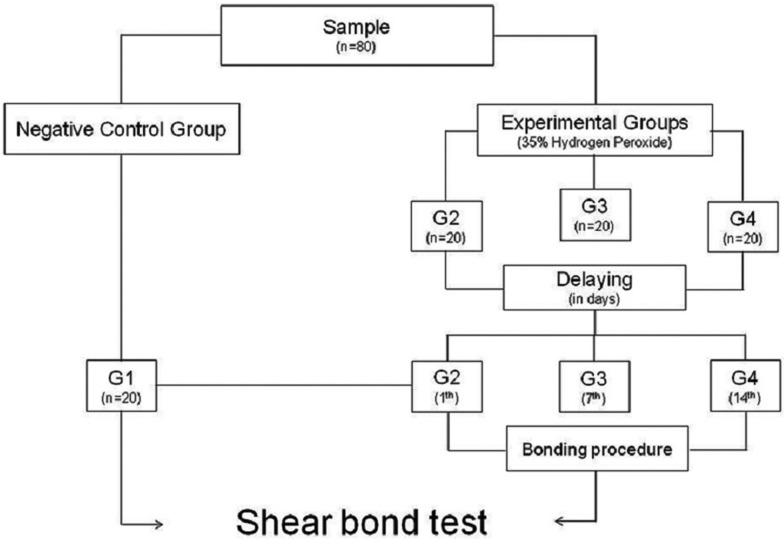
Experimental design study.

Eighty freshly extracted bovine incisors were selected for the study. All teeth had a fully formed apex and a labial surface that was free of fractures, cracks, or erosion. After cleaning, the teeth were sectioned into 5.0-mm segments at the cementoenamel junction. They were embedded in acrylic resin (JET Classico, São Paulo, SP, Brazil) by using a matrix from poly-vinyl chloride tubes (Tigre®, Pouso Alegre, MG, Brazil). The coronary portion of each specimen was polished with pumice (Maquira®, Maringa, PR, Brazil) and water, by using a Robson brush at low speed. Teeth were placed in saline solution for rehydration until use. Materials used in the tooth whitening and bonding procedures are presented in  Table 1. Teeth were randomly divided into four groups (n = 20): G1 (negative control) and G2, G3, and G4 (experimental groups) (Fig. 1).

-Dental bleaching protocol

**Table 1:**
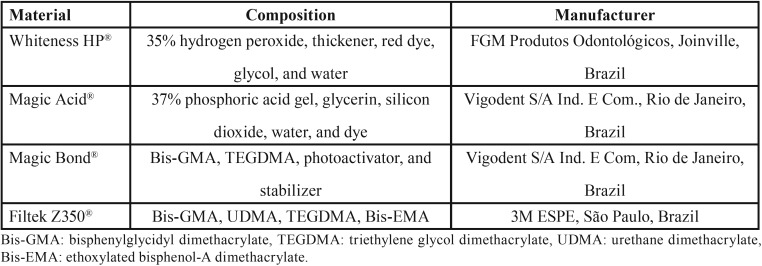
Composition and manufacturer of each material used.

The negative control group (G1) received no bleaching treatment. Experimental groups (G2, G3 and G4) were subjected to bleaching with hydrogen peroxide (35% Whiteness HP®) at a 1:3 ratio of thickener to 35% hydrogen peroxide, according to the manufacturer’s recommendations. The mixture was placed on the buccal surface of each specimen. After 1 minute, photoactivation was performed in three 15-minute periods. The gel was aspirated, and the tooth surface was rinsed with water. Specimens were kept in saline solution until the adhesive procedure was performed 1 day (G2), 7 days (G3) or 14 days (G4) after bleaching.

-Bonding procedure

After the experimental period, teeth were again polished as described above. A 12-mm2 area at the approximate center of the tooth crown was etched with 37% phosphoric acid (Magic Acid®) for 30 seconds, followed by rinsing under running water for 30 seconds. A thin layer of adhesive (Magic Bond®) was applied with a microbrush and light-cured for 20 seconds. A thin layer of resin composite (Z350®) was inserted on the base (11.7 mm2) of a straight-wire-type bracket (Roth Light, Dental Morelli® Ltda., Jundiaí, SP, Brazil). To standardize the adhesive bonding procedure, 300 N of pressure (measured by a tensiometer) was applied to the bracket by pliers, followed by light-curing for 40 seconds. After bonding, all teeth were stored in saline solution for 24 hours until the shear test.

-Shear test

The shear test was carried out in an universal mechanical testing machine (Instron 3342, Canton, MA) by using a load cell of 500 N and a speed of 0.5 mm/min, according to ISO TR11405 for metallic devices. The machine recorded the maximum load at the time of fracture (recorded in N and later transformed into MPa).

-Failure mode analysis

The fracture pattern was analyzed by using a stereomicroscope (Kozo and Electronical Optical Instrumentation, Nanjing, Jiangsu, China) with a magnification of 30x. The adhesive remnant index (ARI) was scored as follows (14): score of 1 = 100% of the adhesive remaining on the enamel; score of 2 = greater than 90% of the adhesive remaining on the enamel; score of 3 = greater than 10% but less than 90% of the adhesive remaining on the enamel; score of 4 = less than 10% of the adhesive remaining on the enamel; and score of 5 = no adhesive remaining on the enamel.

-Statistical analysis

SBS values were analyzed by using one-way analysis of variance (ANOVA) followed by the Tukey test. ARI scores were compared by the Kruskal-Wallis test. All tests were performed in the SPSS for Windows (version 16.0) software package. The significance level was set at 5%.

## Results

Data for the shear bond strength test (MPa) and mode of failure (ARI) are summarized in  Table 2.

**Table 2:**
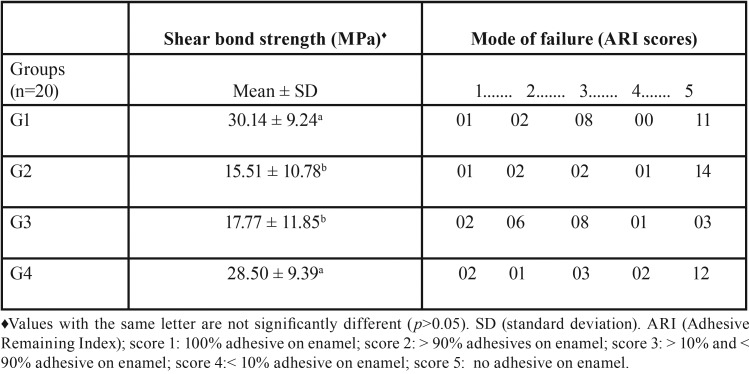
Shear bond strength of adhesive system to enamel and mode of failure.

There was a significant difference between the means of the bond strength of G1 and G2 (*p* <0.001), G1 and G3 (*p* = 0.02) and G3 and G4 (*p* = 0.009).

## Discussion

The null hypothesis was rejected, because the delay had influence on the bond strength of the interface adhesive system/enamel. Average SBS values of the group with bonding 1 day after bleaching (G2) were significantly lower than those of the unbleached control group (G1), consistent with results obtained in earlier studies (6,15-18). When the interval between bleaching and bonding was 7 days (G3), the SBS values were significantly lower compared to those of the unbleached control group (G1). These findings are consistent with the study of Mullins *et al.* (18), who advised that brackets be bonded 14 days after tooth whitening. However, other researchers using similar methodologies (6,19) did not find any significant in SBS values for brackets bonded at the same time intervals after bleaching. One possible reason for this difference may be the use of different materials or methodologies, such as intracoronary bleaching and antioxidant treatment (6,20).

Higher SBS values were found in G4 (bonding 14 days after bleaching), compared to G2 and G3, but not compared to G1. A delay of 7 days was insufficient to decrease the harmful effects of free oxygen from 35% hydrogen peroxide, whereas a delay of 14 days ensured acceptable safety to perform bracket bonding (15,22). However, Uysal *et al.* (21) and Bishara *et al.* (22) suggest that there was no significant reduction in the bond strength, regardless of the time since the whitening procedure. It is relevant to emphasize that the present study used tensiometer which standardized the force applied in each bracket during bonding to the enamel, thereby differentiating of other studies that had not quantified this pressure (5,9).

For a good clinical performance, the mean adhesive strength of a material used for orthodontic appliances bonding to the enamel must be between 5.6 MPa to 7.8 MPa (23). All the groups in this study had values beyond this average. Therefore, acceptable for the orthodontic treatment.

Bond failure may occur on the surface of the bracket, the surface of the tooth or within the adhesive layer. Adhesive fractures occur at the interface of the adhesive with the bracket or enamel, whereas cohesive fractures occur inside the adhesive layer (24,25). In this study only adhesive failures were observed, probably due to bracket characteristic with a metal mesh in the base, which does not allow the adhesion between the resin/bracket interface, but only a mechanical retention on the metal mesh. The metal mesh is a ubiquitous feature of orthodontic brackets, which must be removed without causing damage to the enamel (26).

Teeth in G2 showed no remaining adhesive on the enamel (score 5), consistent with the findings reported by Patusco *et al.* (18). There was no significant difference in the ARI score distribution between G1 and G4, and the most prevalent ARI score of teeth in both groups was a score of 5. Teeth in all groups failed the SBS test, because the main purpose was to determine mode of failure (i.e. bracket debonding). Therefore, it cannot be concluded that failure necessarily occurred due to the effects of whitening. Considering the high ARI scores of teeth in G1 and G4, as well as the ideal strength values for orthodontic treatment (23), debonding in G1 and G4 was likely due to the natural limits of some property of a material involved. For example, one variable involved in the bonding that was not analyzed in this study was the flexural strength of the resin (27). It is possible that teeth in G1 and G4 exhibited deflection of the resin or the bracket at the moment of debonding due to good mechanical retention of the resin to the metal mesh of the bracket base.

It is also important to emphasize that the direction of the force used for orthodontic bracket removal in the clinic is entirely different from the direction used in the shear test. In the mechanical test, the force was applied in the occlusal/gingival direction. In the office, the bracket is removed by a special apparatus in a perpendicular direction to the long axis of the tooth. The clinical approach maintains as much adhesive on the tooth as possible, to ensure greater control of the bracket removal process and less damage to the enamel (26).

So, as conclusion, considering the limitations of current study, bonding of the enamel/adhesive/composite/bracket system was more effective when performed 14 days after bleaching with 35% hydrogen peroxide, compared to shorter time points after bleaching.
